# Cluster sets lead to better performance maintenance and minimize training-induced fatigue than traditional sets

**DOI:** 10.3389/fspor.2024.1467348

**Published:** 2024-12-09

**Authors:** José Antonio Páez-Maldonado, Pedro Jesús Cornejo-Daza, Juan Sánchez-Valdepeñas, Miguel Sánchez-Moreno, Francisco Piqueras-Sanchiz, Manuel Ortega-Becerra, Fernando Pareja-Blanco

**Affiliations:** ^1^ University of Osuna (Centre Attached to the University of Seville), Osuna, Spain; ^2^ Science-Based Training, Physical Performance & Sports Research Center (CIRFD), Universidad Pablo de Olavide, Seville, Spain; ^3^ Department of Sports and Computers Sciences, Faculty of Sport Sciences, Universidad Pablo de Olavide, Seville, Spain; ^4^ Department of Human Movement and Sport Performance, University of Seville, Seville, Spain; ^5^ Physical Education and Sports Department, Cardenal Spínola CEU Andalucía University, Bormujos, Seville, Spain; ^6^ Department of Physical Education and Sports, University of Seville, Seville, Spain

**Keywords:** resistance training, velocity-based training, lactate, electromyography, tensiomyography

## Abstract

**Objective:**

The aim of this study was to examine the acute effects on mechanical, neuromuscular, metabolic, and muscle contractile responses to different set configurations in full-squat (SQ).

**Methods:**

Twenty-two men performed three SQ sessions that consisted of 3 sets of 12 repetitions with 60% 1RM with 4 minutes inter-set rests: a) traditional set (TS): no rest within the set; b) cluster-6 (CS6): a 30 seconds intraset rest after the 6th repetition of each set; and c) cluster-2 (CS2): a 30 seconds intraset rest every 2 repetitions. Mechanical (i.e., force, velocity, and power) and electromyography (EMG) values were recorded for every repetition. A battery of tests was performed: a) tensiomyography (TMG), b) blood lactate c), countermovement jump (CMJ), d) maximal isometric SQ, and e) performance with the load that resulted in a velocity of 1 m·s−^1^ at baseline (V1-load). Repeated measured ANOVA analyses were used to compare the 3 protocols.

**Results:**

As the number of intraset rests increased (TS < CS6 < CS2), mechanical performance was better maintained (*p* < 0.01) and EMG variables were less altered (*p* = 0.05). At post, CS2 and CS6 displayed lower lactate concentration, lesser reductions in CMJ height, and smaller alterations in TMG-derived variables than TS (*p* < 0.05).

**Conclusion:**

The introduction of short and frequent intraset rest periods during resistance exercise alleviates training-induced fatigue, resulting in better maintenance of performance. This approach can be applied during the in-season period when minimizing fatigue is a priority.

## 1 Introduction

In resistance training (RT) settings, traditional sets (TS) have been prescribed by performing consecutive repetitions without rest between them, and with inter-set rest periods (1, 2). However, as the number of repetitions increases within the TS configuration, it leads to higher fatigue development, causing declines in force production, and, as a result, decreased barbell velocity and power output (3, 4). Although certain fatigue development is required to maximize strength gains (5), minimizing RT-induced fatigue may be beneficial in certain contexts. Cluster training (CS) introduces brief rest periods, typically 10–45 s, either between repetitions or blocks of repetitions (6, 7). This approach is effective at maintaining performance during RT sessions (8–11). Notably, CS displays reduced muscle damage, blood lactate concentrations, and hormonal responses compared to TS with workload-matched protocols (11, 12). The inclusion of rest intervals within sets during CS is believed to contribute to the recovery of bioenergetic components like phosphocreatine (PCr) and adenosine triphosphate (ATP) (13). Consequently, CS could emerge as an advantageous strategy as it enables the completion of the same workload with less fatigue development (2).

Electromyography (EMG) may provide a better understanding of the mechanisms behind the changes in mechanical performance, such as muscle activation and neuromuscular fatigue accumulated throughout the training session, which allows researchers and coaches to quantitatively assess the neuromuscular behavior during specific tasks (14). Despite its significance, research exploring the effects of various set configurations on EMG activity remains limited. To date, only one study has delved into the impact of CS configuration on EMG activity during lower-body exercises (15). In this particular study, two conditions were compared: 6 sets of 6 reps at 20% 1RM in the loaded countermovement jump (CMJ) exercise either continuously (i.e., TS) or with a 30-s pause every 2 repetitions (i.e., CS) (15). Their findings revealed that the CS configuration led to greater root mean square (RMS) values in the vastus lateralis (VL) and rectus femoris muscles when compared to the TS configuration, but not in the vastus medialis (VM) (15). Additionally, a progressive decline in median frequency (MDF) over time was noted, although no differences were observed between the two set configurations (15).

Tensiomyography (TMG) is an effective technique used for assessing passive muscle contractile properties *in vivo* by measuring how muscle bellies respond in terms of time and radial deformation to a single twitch stimulus (16). TMG is commonly used in evaluating changes in muscle contractile properties resulting from fatigue induced by RT sessions (17, 18). Among the main parameters obtained from a TMG assessment highlight: maximum radial displacement (Dm), contraction time (Tc), delay time (Td), and velocity of muscle deformation (Vd) (19). Dm is defined as the transverse deformation of the muscle (20). A long-term reduction in Dm is interpreted as an increase in muscle stiffness, and vice versa (20); however, Dm may also acutely decrease due to exercise-induced fatigue (21). Td represents the time it takes for the analyzed muscle structure to reach 10% of the total displacement observed after stimulation (10% of Dm). Naturally, its value depends on the dominant fiber type in the muscle, its fatigue state, and its level of potentiation and activation (22). Tc is the time that elapses from Td (10% of Dm) until 90% of Dm is reached. A strong relationship has been observed between Tc and fiber type distribution (23). Likewise, Vd reduction indicates muscle fatigue, meaning a decline in the rate at which the muscle contracts in response to a stimulus (21). The fact that TMG analyzes muscle function non-invasively and selectively is especially valued by coaches, and sports scientists, who prefer precise and practical assessment methods that do not disrupt their professional routines. However, there is limited research on the acute effects of different set configurations on TMG (19, 20). One study highlighted greater impairments in Vd following a protocol consisting of 3 × 8 with 75% 1RM in full-squat (SQ) compared to the 6 × 4 protocol (20).

Although CS is widely used in training, there is a lack of understanding of how different set configurations, such as CS and traditional sets, impact neuromuscular responses and fatigue. Current assessments like EMG and TMG are underutilized in evaluating these configurations, creating a knowledge gap. Addressing this is critical for optimizing training outcomes, ensuring athlete safety, and enabling personalized adaptations. Therefore, the purpose of this study was to analyze the acute integral response, including mechanical, neuromuscular, metabolic, and muscle contractile properties during and following different SQ set configurations. We hypothesized that CS would better preserve mechanical performance compared to TS, due to the reduced fatigue associated with CS.

## 2 Materials and method

### 2.1 Study design

**Figure 1:**
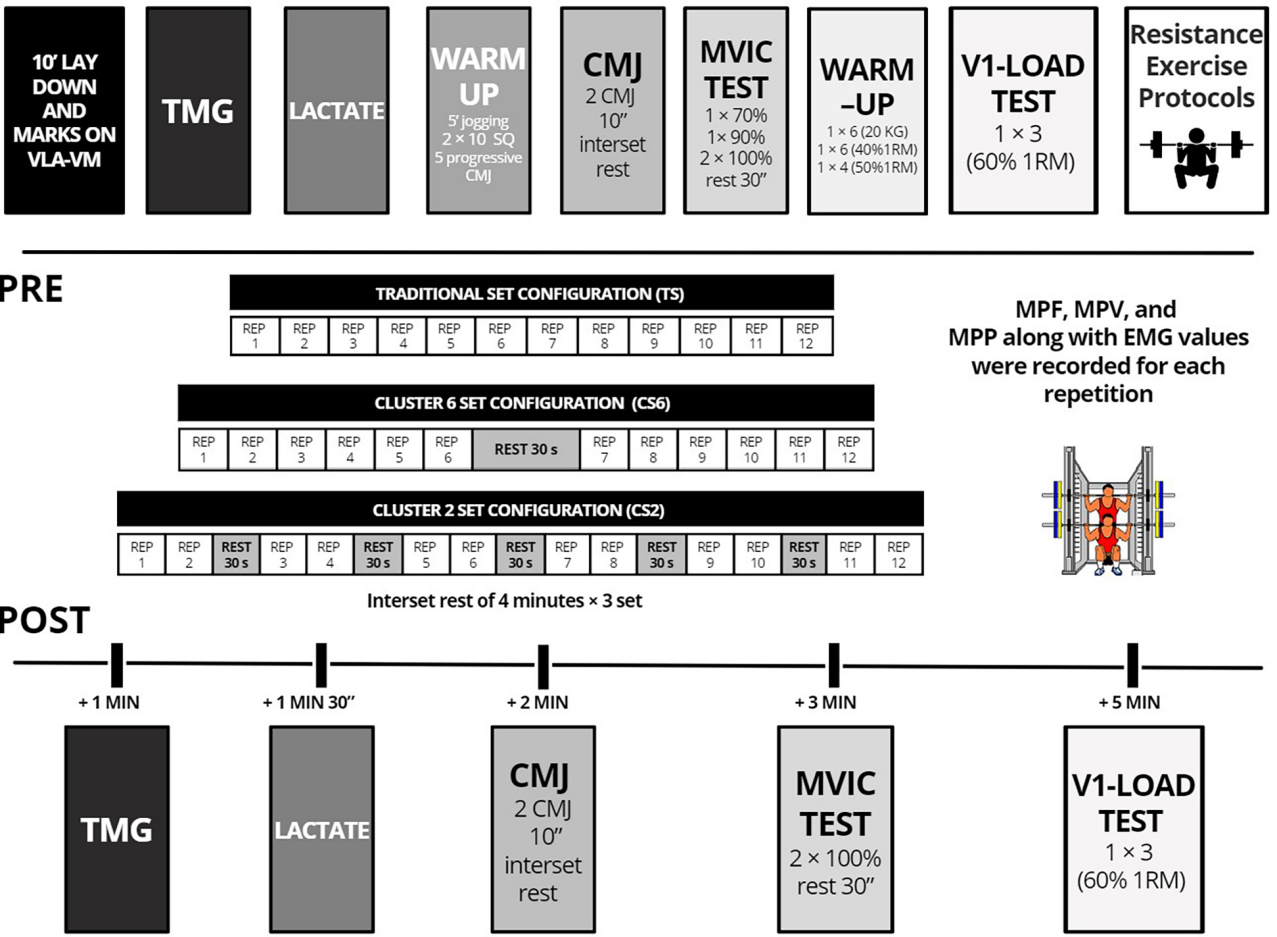
Schematic representation of the study design for the resistance exercise protocols analyzed and the timeline of the battery of tests conducted before and after the protocols. CMJ, countermovement jump; TMG, tensiomyography; SQ, full-squat; MVIC, maximal voluntary isometric contraction; V_1_-load: test against the load that elicited a ∼1 m·s^−1^ velocity at baseline SQ measurements. EMG, kinetic, and kinematic data were recorded during the training sets.

A randomized cross-over within-participant design was implemented to explore the acute responses in mechanical performance, neuromuscular activity, muscle mechanical properties, and metabolic stress to three resistance exercise protocols differing in the set configuration in a Smith Machine: (a) traditional set (TS): no rest within the set; (b) cluster-6 (CS6): a 30 s intraset rest after the 6th repetition of each set; and (c) cluster-2 (CS2): a 30 s intraset rest every 2 repetitions. The relative intensity (60% 1RM), volume (3 sets of 12 repetitions), between-set rest time (4 min), and exercise (SQ) were matched between protocols. The protocols were separated by 1 week, with a random order of presentation. A battery of tests was performed before (Pre) and after (Post) each protocol in this order: (a) blood lactate, (b) TMG (c) CMJ; (d) maximal voluntary isometric contraction (MVIC) in 90° SQ, and (e) performance with the load that elicited a ∼1 m·s^−1^ velocity at baseline measurements (V1-load) in SQ (Figure 1). Furthermore, kinetic, kinematic, and EMG data were recorded for every repetition. One week preceding the beginning of the study a loading SQ test was conducted to establish the individual load-velocity relationship for each subject. Protocols were conducted in a research laboratory under the direct supervision of researchers, with the same environmental conditions (20 °C and 60% humidity), and at the same time of day (±1 h) to avoid likely inferences with circadian cycles. Participants received strong verbal encouragement to exert their maximum effort during all protocols.

### 2.2 Participants

Sample size was calculated using GPower (version 3.1.9.4, Düsseldorf, Germany) (24) introducing the following parameters: a 3 (protocol) × 2 (Pre vs. Post) repeated measures ANOVA, expected effect size (ES) between protocols (0.5), error probability (0.05) and power (0.95), which resulted in a sample size of 18 participants. For that, we decided to recruit 22 moderately resistance-trained men (age 25.3 ± 4.4 years; height 1.77 ± 0.08 m; body mass 75.4 ± 8.5 kg; relative 1RM SQ = 1.37 ± 0.19 kg per body mass, from 1.5 to 4 years of RT background) assuming the likely loss of data in some of the analyzed variables. Exclusion criteria included presenting any physical limitation, health problem, or musculoskeletal injury that could affect their performance in the tests. Participants received information regarding the procedures, potential benefits, and associated risks before providing written informed consent. Participants were instructed to maintain their regular diet and refrain from engaging in strenuous physical activity for 72 h before each protocol. The study was approved by the Research Ethics Committee (Ref: 03-819) and adhered to the guidelines outlined in the Declaration of Helsinki.

### 2.3 Testing procedures

#### 2.3.1 Progressive loading test

A progressive loading test was performed using a Smith machine (Multipower Fitness Line, Peroga, Murcia, Spain) to determine the individual load-velocity relationships in the SQ exercise. The SQ was performed with participants starting from the upright position with the knees and hips fully extended and stance approximately shoulder-width apart, and the barbell resting across the back at the level of the acromion. The movement involved descending at a controlled mean velocity (∼0.50–0.65 m·s^−1^) as low as possible (∼35–40° knee angle), followed by an immediate return to the upright position (full knee extension 180°). Unlike the eccentric phase, participants executed the concentric phase at their maximal intended velocity. Velocity data were recorded with a linear velocity transducer (T-Force System Ergotech, Murcia, Spain). The test commenced with a 20 kg load, progressively increasing in 10 kg increments. When the mean propulsive velocity (MPV) dropped below 0.60 m·s^−1^, increments were adjusted to 5 kg until the MPV fell below 0.50 m·s^−1^. For light loads, (≥1.00 m·s^−1^) three repetitions were executed, two for medium loads (1.00–0.80 m·s^−1^), and one for the heavier loads (≤ 0.80 m·s^−1^), with 3 min of recovery between sets. The repetition achieving the highest MPV for each load was selected for subsequent analysis. MPV corresponds to the portion of the concentric phase during which the measured acceleration exceeds the acceleration due to gravity (‒9.81 m·s^−2^) (25). On average, the participants completed 8.9 ± 1.2 loads during the test.

#### 2.3.2 Blood lactate concentration

Lactate was measured using a portable lactate analyzer (Lactate Pro 2, Arkray, Kyoto, Japan) following the manufacturer's indications. This system has shown high reliability for a physiological range of 0.5–25.0 mmol·L^−1^ (26). Blood capillary samples were obtained from the middle finger.

#### 2.3.3 Tensiomyography

TMG has been validated for the assessment of *in vivo* passive muscle contractile properties in response to single-twitch stimulation (27). The VL contractile properties of the left leg were evaluated using a TMG (TMG-100 system electro-stimulator, TMG-BMC, Ljubljana, Slovenia). The electric stimulus was evoked through two self-adhesive electrodes (5 cm × 5 cm, Dura-Stick® premium, Cefar-Compex, Hanover, Germany) separated by 5 cm on the VL muscle of the left leg following SENIAM indications (28). The muscle contractile response was assessed with a digital Dc-Dc transducer Trans-TekR (GK 40, Ljubliana, Slovenia) located perpendicular to the muscle belly and at an equal distance from the self-adhesive electrodes. Measurements were acquired in a supine position and the left knee joint was fixed at an angle of ∼140° using a wedge cushion located below the popliteal fossa. Electrical stimulation was applied with an initial amplitude of 40 mA and a pulse duration of 1 ms, increasing 10 by 10 mA every 10 s until the maximum output of the stimulator (100 mA) (29). The following variables were examined in the present study: Tc, Dm, Td, and Vd. Dm was defined as the peak amplitude in the displacement-time curve of the twitch response; Tc was obtained by determining the time interval from 10% to 90% of Dm; Td was defined as the time between the electrical stimulus and 10% of Dm (19); and Vd was calculated as Dm · (Tc + Td)^−1^ (30, 31). All measurements were carried out by the same investigator and the curve with the highest Dm value was considered for further analysis.

#### 2.3.4 Countermovement jump

Jump height was measured using an infrared timing system (OptojumpNext, Microgate, Bolzano, Italy) previously validated (32). Three attempts with a 20 s rest were performed and the average height was recorded for further analyses. Participants were instructed to maintain both hands resting on the waist and try to attain their maximal vertical height after a fast downward movement close to 90° of knee flexion. All participants were instructed to land in an upright position and to bend their knees after landing. The warm-up consisted of jogging for 5 min, 2 sets of 10 SQ without external load, and 5 plus 2 submaximal CMJs.

#### 2.3.5 Maximal voluntary isometric contraction test

This test was performed on a Smith machine with height-adjustable movable supports to standardize the individual test position, which was established at 90° knee flexion in SQ. Participants were instructed to push as fast and hard as possible for 5 s after the cue “Ready, set, go!” Two attempts, separated by a 1 min rest, were performed. EMG data were recorded as described below. Kinetic data were collected at a sampling rate of 1,000 Hz with an 80 × 80-cm dynamometric platform (FP-500, Ergotech, Murcia, Spain). Raw force-time data were automatically processed (4th order low-pass Butterworth filter with no phase shift using a 200 Hz cut-off frequency) with the custom software (T-Force System, Ergotech). Maximal isometric force (MIF), maximal rate of force development (RFDmax), which was established as the maximum slope in the force-time curve with 20 ms time intervals, and the average tangential slope of the force-time curve obtained over different time intervals (50, 100, 150, 200 and 400 ms) from the onset of force production (RFD_0–50_, RFD_0–100_, RFD_0–150_, RFD_0–200,_ and RFD_0–400_, respectively) were subsequently calculated. The onset of the force signal was established when the values were raised above 2 standard deviations (SDs) from the baseline signal. The average value of each variable in the two attempts was recorded for further analysis. The warm-up consisted of 2 attempts at 70% and 90% of the perceived effort with 30 s rest between them.

#### 2.3.6 V1-load test

This test consisted of performing 3 SQ repetitions with the V1-load (∼60% 1RM), which was the load that elicited a 1 m·s^−1^ at the Pre-test (33). The execution technique is described in the “Progressive loading test” section. Mean propulsive force, velocity, and power (MPF, MPV, and MPP) were recorded with a linear velocity transducer synchronized with a dynamometric platform (T-Force System Ergotech, Murcia, Spain). The highest value of each variable was used for further analysis. EMG data were recorded as described below. The warm-up consisted of 6-6-4 repetitions with 20 kg, 40%, and 50% 1RM, respectively, with a 3-min rest between sets.

#### 2.3.7 EMG signal acquisition

After skin preparation and following the SENIAM recommendations (28), surface EMG electrodes were placed on the right leg of the VM and VL muscles. A parallel-bar, bipolar surface electromyographic sensor Trigno^™^ wireless EMG system (Delsys, Inc., Natick, MA, USA), with an interelectrode distance of 10 mm, common mode rejection ratio >80 dB, and bandwidth filter between 20 and 450 Hz ± 10% was used to measure EMG signal. The baseline noise was <5 µV peak-to-peak and the sampling rate was 2,000 Hz. The raw data from the EMG were stored in digital format using EMG Works Acquisition software (Delsys, Inc, MA, USA). From each contraction, the highest (over sliding windows of 500 ms with an overlap of 499 ms) root mean square (RMS) and median frequency (MDF) values for each muscle were recorded. RMS and MDF values were averaged from both muscles in each repetition for further analysis. EMG signal was recorded for every repetition during RT protocols, MVIC, and V1-load tests. The signal from MVIC at the Pre-test of each protocol was used to normalize the EMG parameter. Therefore, RMS and MDF obtained from MVIC at Pre-test were 100%.

#### 2.3.8 Resistance exercise protocols

All participants remained in the lying position for 10 min before starting the baseline data acquisition (blood sample and TMG measurement) to minimize the effects of any previous activity. While participants were in a supine position, electrode locations for TMG and EMG were marked. Then, blood lactate, TMG, CMJ, MVIC, and V1-load tests were performed (Pre). After taking baseline values, the RT protocol was performed. The SQ execution technique in all protocols was the same as described in the “Progressive Loading test” section. All participants performed on a Smith machine three SQ sessions matched in intensity (60% 1RM), volume (3 sets of 12 repetitions), and inter-sets rest time (4 min). The independent variable was the set configuration: (a) TS: no rest within the set; (b) CS6: a 30 s intraset rest after the 6th repetition of each set; and (c) CS2: a 30 s intraset rest every 2 repetitions (i.e., after the 2nd, 4th, 6th, 8th, and 10th repetition of each set). Relative loads were determined MPV at which every%1RM was attained, which was obtained from the individual second-order load-velocity relationship (*R*^2^ = 0.996 ± 0.004) derived from the progressive loading test. The absolute loads (in kg) were individually adjusted in every session to the corresponding MPV matched (±0.03 m·s^−1^) associated with the prescribed%1RM. We used a range of 0.03 m·s^−1^ since it has been shown that this value is the smallest detectable change in MPV when using the T-Force System in the SQ exercise on a Smith machine (34). A force platform (FP-500, Ergotech, Murcia, Spain) synchronized with a linear velocity transducer (T-Force System, Ergotech, Murcia, Spain) was installed on the Smith machine to record MPF, MPP, and MPV for each repetition. Besides, EMG data were recorded throughout the 36 repetitions. After the last repetition of the third set, the battery of tests was repeated at Post as follows: (1) TMG (at 60 s); (2) blood lactate (at 90 s); (3) CMJ (at 120 s); (4) MVIC (at 180 s) and (5) V1-load (at 300 s).

### 2.4 Statistical analyses

Values are reported as mean ± SD. The Shapiro–Wilk test of normality was conducted to ensure normal data distribution at Pre. Test–retest reliability was measured by the standard error of measurement (SEM; root mean square of the intrasubject total mean square), which was expressed in relative terms through the CV. Relative reliability was calculated by the intra-class correlation coefficient with 95% confidence intervals [ICC (95% CI)], which was calculated with the one-way random effects model. CV and ICC were obtained from the three baseline values obtained from each condition. The reliability of EMG values was calculated from the MVIC test. A one-way repeated measures analysis of variance (ANOVA) was used to compare the average values achieved during each protocol. A 3 (protocol) × 2 (Pre vs. Post) repeated measures ANOVA was performed to analyze the acute responses to each protocol. A 3 (protocol) × 36 (repetitions) repeated measures ANOVA was conducted to compare the differences between protocols in the various repetitions. Bonferroni's *post hoc* comparisons were used as necessary, and statistical significance was set at *p* ≤ 0.05. Pre-post effect size (ES) values were calculated using Hedge's *g* on the pooled SD (35). The ES of *post hoc* comparisons was calculated using Cohen's *d*, which was interpreted as a low (<0.50), moderate (0.50–0.79), or large effect (>0.80) (36). For CV, values below 10% were considered acceptable, indicating low variability, and ICC) values were interpreted as follows: poor reliability (<0.5), moderate reliability (0.5–0.75), good reliability (0.75–0.9), and excellent reliability (>0.9) (37). All statistical analyses were performed using SPSS version 25.0 software (SPSS, Inc., Chicago, IL, USA), in addition to Microsoft Office Excel 2007 for calculating ES and CV.

## 3 Results

The reliability values of the different tests conducted are shown in Table 1.

**Table 1:** Reliability at baseline of the different variables under study.

	ICC (95% CI)	CV (%)
TMG variables		
Dm	0.98 (0.97–0.99)	6.1%
Tc	0.94 (0.90–0.96)	5.1%
Td	0.97 (0.95–0.98)	2.1%
Vd	0.99 (0.98–0.99)	5.7%
CMJ	0.99 (0.98–0.99)	2.1%
MVIC variables		
MIF	0.95 (0.91–0.97)	5.9%
RFDmax	0.91 (0.80–0.96)	8.1%
RFD_0–50_	0.77 (0.40–0.91)	23.8%
RFD_0–100_	0.76 (0.19–0.93)	21.4%
RFD_0–150_	0.82 (0.55–0.93)	18.2%
RFD_0–200_	0.74 (0.46–0.97)	14.5%
RFD_0–400_	0.74 (0.47–0.87)	12.2%
V1-load variables		
MPF	0.98 (0.97–0.99)	3.6%
MPV	0.88 (0.82–0.92)	5.3%
MPP	0.89 (0.84–0.93)	7.5%
EMG variables		
RMS	0.98 (0.97–0.99)	9.3%
MDF	0.96 (0.93–0.97)	5.2%

ICC, intraclass correlation coefficient; CI, confidence interval; CV, coefficient of variation; TMG, tensiomyography; Dm, muscle displacement; Tc, contraction time; Td, delay time; Vd, velocity of deformation; CMJ, countermovement jump; MVIC, maximal voluntary isometric contraction; MIF, maximal isometric force; RFDmax, maximal rate of force development; RFD_0–50_, rate of force development from the onset of force production to 50 ms; RFD_0–100_, from the onset of force production to 100 ms; RFD_0–150_, from the onset of force production to 150 ms; RFD_0–200_, from the onset of force production to 200 ms; RFD_0–400_, from the onset of force production to 400 ms; V1-load, load representing the 60% 1RM at pre-testing; MPF, mean propulsive force; MPV, mean propulsive velocity; MPP, mean propulsive power; RMS, root mean square averaged from the vastus medialis and vastus lateralis muscles; MDF, median frequency averaged from the vastus medialis and vastus lateralis muscles.

### 3.1 Descriptive characteristics of the resistance exercise protocol

According to the schedule, no differences between protocols were observed in the fastest velocity at which the absolute load was lifted (*p* = 0.51). There was a significant “protocol” effect for the rest of the variables analyzed, except for RMS (Table 2). The velocity loss within the set increased as the number of intraset rest periods decreased (TS > CS6 > CS2). MPV and MPP progressively increased as the number of intraset rests increased (TS < CS6 < CS2). Likewise, CS2 showed greater MPF (*p* < 0.05) than TS. MDF decreased as decreased the number of intraset rest (TS < CS6 < CS2).

**Table 2:** Mechanical and neuromuscular characteristics of each resistance exercise protocol (average of 36 repetitions).

	TS	CS6	CS2	Protocol *p*-value
MPV-best (m·s^−1^)	0.92 ± 0.09	0.92 ± 0.09	0.92 ± 0.10	*F* = 0.69; *p* = 0.51
MPV-loss (%)	31.5 ± 10.4^CS6, CS2^	28.0 ± 9.8^CS2^	17.3 ± 7.1	*F* = 86.3; *p* < 0.001
MPV (m·s^−1^)	0.77 ± 0.11^CS6, CS2^	0.80 ± 0.10^CS2^	0.84 ± 0.10	*F* = 96.1; *p* < 0.001
MPF (N)	677.3 ± 91.0^CS2, CS6^	687.3 ± 90.8^CS2^	699.8 ± 105.0	*F* = 17.8; *p* *<* 0.001
MPP (w)	498.5 ± 88.0^CS6, CS2^	518.1 ± 76.8^CS2^	551.4 ± 88.2	*F* = 60.7; *p* < 0.001
RMS (%)	114.2 ± 25.6^CS2^	101.6 ± 14.7	97.4 ± 15.1	*F* = 5.14; *p* *=* 0.16
MDF (%)	85.0 ± 10.3^CS6, CS2^	93.0 ± 14.5^CS2^	102.5 ± 10.8	*F* = 20.4; *p* < 0.001

Data are mean ± SD, *n* = 22. TS, traditional sets; CS6, cluster sets of 6 repetitions; CS2, cluster sets of 2 repetitions; MPV-best, best mean propulsive velocity (MPV) within the training session; MPV-loss, averaged velocity loss within each set calculated as the relative difference between the best and the last MPV within each set; MPF, mean propulsive force; MPP, mean propulsive power; RMS, root mean square from electromyography (EMG) data; MDF, median frequency from EMG data. Statistically significant differences with a CS2 protocol: CS2 (*p* < 0.05). Statistically significant differences with a CS6 protocol: CS6 (*p* < 0.05).

**Figure 2:**
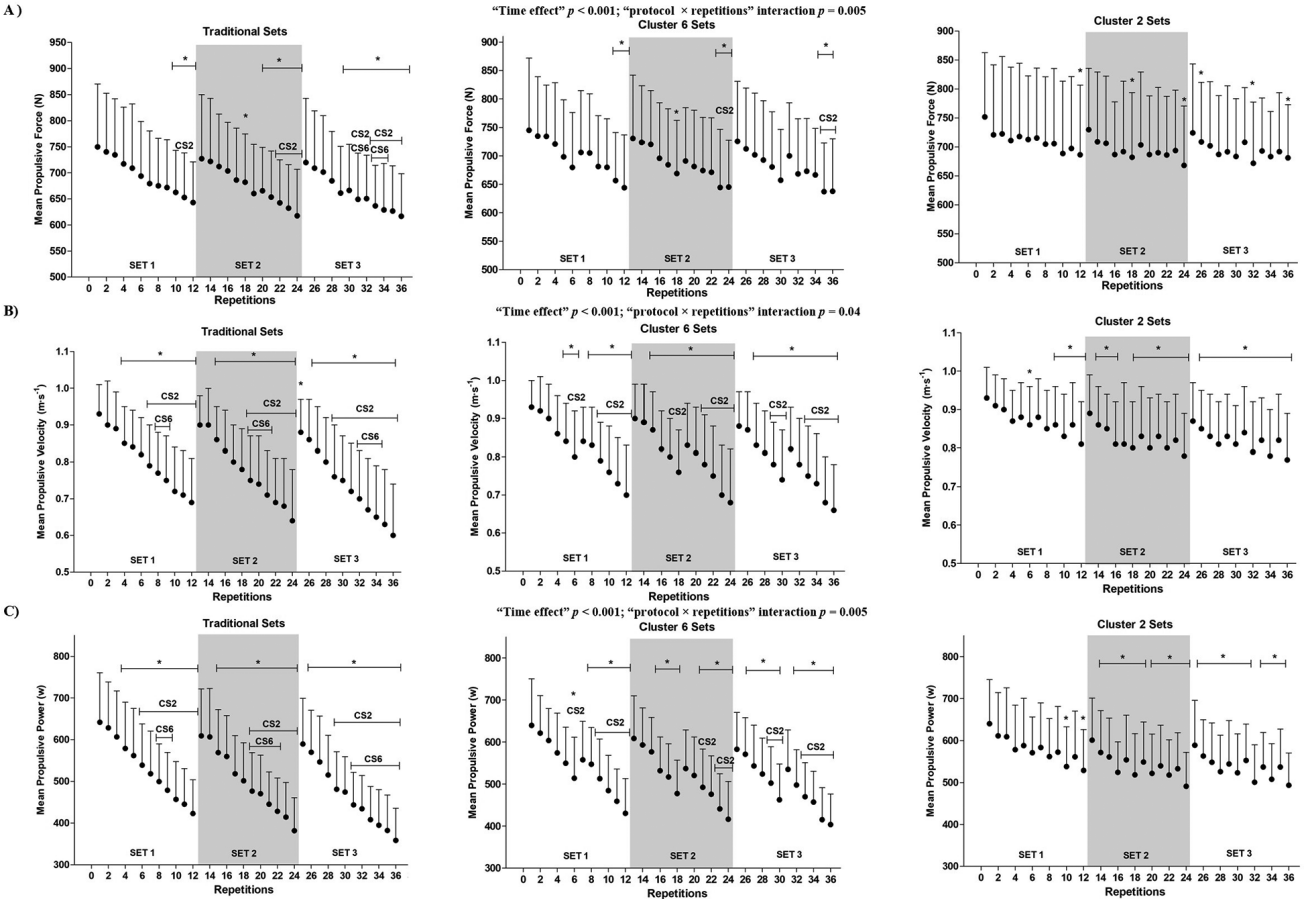
Evolution of mechanical parameters throughout the 36 repetitions for each resistance exercise protocol. **(A)** mean propulsive force; **(B)** mean propulsive velocity; and **(C)** mean propulsive power. Data are expressed as mean ± *SD* (*N* = 22). CS2 indicates significant differences with the CS2 protocol (i.e., cluster sets of 2 repetitions) at the corresponding time point (*p* < 0.05). CS6 indicates significant differences with the CS6 protocol (i.e., cluster sets of 6 repetitions) at the corresponding time point (*p* < 0.05). Statistically significant differences with repetition 1 at the corresponding protocol and time point: *p < 0.05.

Regarding the evolution of performance (MPF, MPV, and MPP) throughout the 36 repetitions, significant “protocol × repetitions” interactions (all *p* < 0.01) were observed (Figure 2). Performance was better maintained as increased the number of intraset rests (TS < CS6 < CS2).

**Figure 3:**
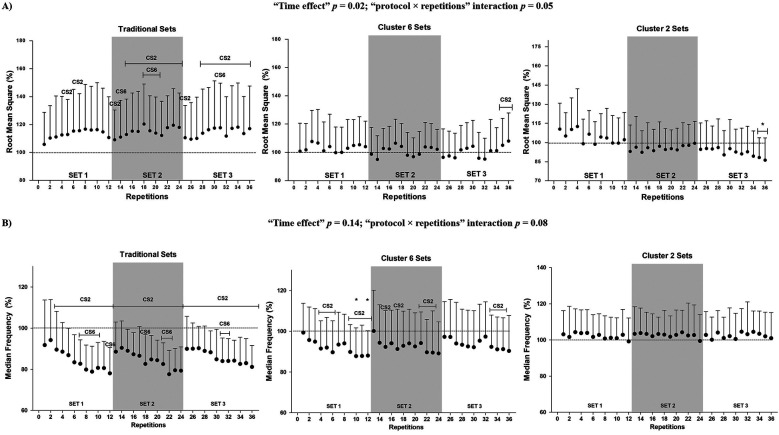
Evolution of neuromuscular parameters throughout the 36 repetitions for each resistance exercise protocol. **(A)** Root mean square averaged from the vastus medialis and vastus lateralis muscles; **(B)** median frequency averaged from the vastus medialis and vastus lateralis muscles. Data are expressed as mean ± *SD* (*N* = 22). CS2 indicates significant differences with the CS2 protocol (i.e., cluster sets of 2 repetitions) at the corresponding time point (*p* < 0.05). CS6 indicates significant differences with the CS6 protocol (i.e., cluster sets of 6 repetitions) at the corresponding time point (*p* < 0.05). Statistically significant differences with repetition 1 at the corresponding protocol and time point: *p < 0.05.

Concerning the evolution of neuromuscular characteristics throughout the 36 repetitions, a significant “protocol × repetitions” interaction was observed for RMS (*p* = 0.05) (Figure 3). TS resulted in significant increases in RMS and decreases in MDF throughout the 36 repetitions, while CS2 maintained the values in these variables without significant changes.

### 3.2 Tensiomyography

Significant “protocol × time” interactions were found for Td, Dm, and Vd (*p* < 0.05) (Table 3). TS and CS6 protocols evoked higher impairments in Dm and Vd than CS2. Furthermore, TS resulted in greater increases in Tc than CS2. Interestingly, a significant reduction in Td was observed for TS and CS6 protocols.

**Table 3:** Effects of different resistance exercise protocols on muscles’ contractile properties assessed by tensiomyography.

	TS			CS6			CS2			ANOVA		
	Pre	Post	ES	Pre	Post	ES	Pre	Post	ES	Time effect	Protocol effect	Protocol × time
Tc (ms)	24.6 ± 3.5	26.6 ± 5.3^CS2^	0.44	24.9 ± 3.8	24.3 ± 2.8	−0.18	24.3 ± 3.7	23.3 ± 3.4	−0.28	*F* = 0.14; *p* = 0.71	*F* = 3.60; *p* = 0.05	*F* = 3.11; *p* = 0.07
Td (ms)	24.3 ± 1.9	22.7 ± 2.5**	−0.66	23.9 ± 2.6	23.0 ± 3.3	−0.30	24.6 ± 2.9	21.9 ± 2.6***	−0.96	*F* = 24.0; *p* < 0.001	*F* = 0.14; *p* = 0.87	*F* = 5.95; *p* = 0.01
Dm (mm)	5.89 ± 1.43	3.59 ± 1.07^***CS2^	−1.78	5.47 ± 1.97	4.26 ± 1.29^**CS2^	−0.71	6.18 ± 2.25	5.69 ± 2.55	−0.20	*F* = 19.3; *p* < 0.001	*F* = 3.70; *p* = 0.04	*F* = 10.2*; p* = 0.001
Vd (mm·ms^−1^)	0.121 ± 0.027	0.074 ± 0.024^***CS2^	−1.81	0.113 ± 0.045	0.090 ± 0.027^*CS2^	−0.61	0.128 ± 0.048	0.128 ± 0.060	0.00	*F* = 11.4; *p* < 0.003	*F* = 4.89; *p* < *0.02*	*F* = 13.9; *p* < 0.001

Data are mean ± SD, *n* = 22. TS: protocol consisted of performing 3 sets of 12 repetitions with 60% of 1RM; CS6: protocol consisted of performing 3 sets of 12 repetitions with 30 seconds intra-set rest after 6 repetitions with 60% of 1RM; CS2: protocol consisted of performing 3 sets of 12 repetitions with 30 seconds intra-set rest after 2 repetitions with 60% of 1RM; Tc, contraction time; Td, delay time; Dm, muscle displacement; Vd, Velocity of deformation radial (Dm/(Tc+Td); ES = within-protocol effect size from Pre to Post. Intra-protocol significant differences from Pre to Post: *p< 0.05, **p< 0.01, ***p< 0.001. ^CS2^indicates significant differences with the CS2 protocol at the corresponding time-point.

### 3.3 Neuromuscular and mechanical response during maximal voluntary isometric contraction

**Figure 4:**
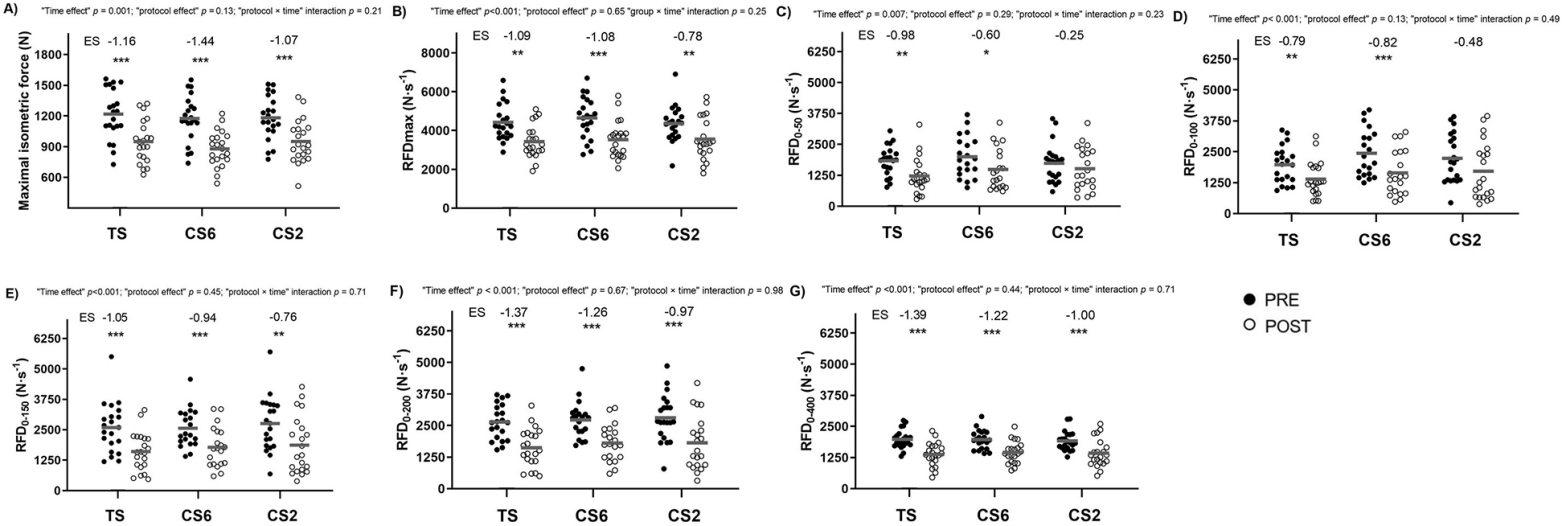
Mechanical responses during the maximal isometric voluntary squat contraction test to the different resistance exercise protocols. **(A)** maximal isometric force; **(B)** maximal rate of force development (RFDmax); **(C)** RFD_0–50_: rate of force development (RFD) from the onset of force production to 50 ms; **(D)** RFD_0–100_: RFD from the onset of force production to 100 ms; (E) RFD_0–150_: RFD from the onset of force production to 150 ms; **(F)** RFD_0–200_: RFD from the onset of force production to 200 ms; and **(G)** RFD_0–400_: RFD from the onset of force production to 400 ms. Data are mean ± SD, *n* = 22. TS, traditional sets; CS6, cluster sets of 6 repetitions; CS2, cluster sets of 2 repetitions; Pre, baseline measure; Post, after exercise; ES, effect size. Intragroup significant differences from Pre- to Post-training: *p *<* 0.05 **p *<* 0.01, ***p *<* 0.001.

No “protocol × time” interactions were noted for any mechanical variable (Figure 4). A “protocol × time” interaction was observed for RMS (*p* = 0.05). CS6 and CS2 protocols elicited significant declines in RMS while TS did not (Table 4).

**Table 4:** Neuromuscular responses during the maximal isometric voluntary squat contraction test to the different resistance exercise protocols.

Neuromuscular response	TS			CS6			CS2			ANOVA		
	Pre	Post	ES	Pre	Post	ES	Pre	Post	ES			
										Time effect	Protocol effect	Protocol × time
RMS (%)	100.0 ± 0	100.0 ± 34.6	0.00	100.0 ± 0	85.7 ± 24.1*	−0.82	100.0 ± 0	78.3 ± 31.3**	−0.96	*F* = 6.10; *p* = 0.02	*F* = 3.60; *p* = 0.05	*F* = 3.60; *p* = 0.05
MDF (%)	100.0 ± 0	96.3 ± 9.0	−0.57	100.0 ± 0	105.5 ± 16.0	0.47	100.0 ± 0	105.5 ± 13.9	0.55	*F* = 2.38; *p* = 0.14	*F* = 2.93; *p* = 0.08	*F* = 2.93; *p* = 0.08

Data are mean ± SD, *n* = 22. TS, traditional sets; CS6, cluster sets of 6 repetitions; CS2, cluster sets of 2 repetitions; RMS, root mean square from EMG data; MDF, median frequency from EMG data; Pre, baseline measure; Post, after exercise; ES, effect size. Intragroup significant differences from Pre- to Post-training: ^*^p< 0.05 **p< 0.01.

### 3.4 Metabolic response, jump performance, and V1-load test

Significant “protocol × time” interactions were observed for blood lactate, CMJ height, and performance against the V1-load (MPF, MPV, and MPP), but not for neuromuscular variables (RMS and MDF) (Table 5). Blood lactate concentration increased as decreased the number of intraset rests (TS > CS6 > CS2). TS resulted in higher impairments of CMJ and MPF, than CS2. Likewise, TS also showed lower MPV and MPP values at Post than CS6.

**Table 5:** Metabolic, mechanical, and neuromuscular response to the different resistance exercise protocols under study.

	TS			CS6			CS2			ANOVA		
	Pre	Post	ES	Pre	Post	ES	Pre	Post	ES	Time Effect	Protocol effect	Protocol × time
Metabolic response												
Lactate (mmol·L^−1^)	1.5 ± 0.4	12.8 ± 3.0^***CS6, CS2^	5.18	1.6 ± 0.4	10.2 ± 4.4^***CS2^	2.70	1.7 ± 0.5	4.1 ± 2.3***	1.41	*F* = 189.1; *p* < 0.001	*F* = 69.2; *p* < 0.001	*F* = 67.8; *p* < 0.001
Mechanical response												
CMJ (cm)	36.1 ± 9.4	27.5 ± 7.3^***CS2^	−1.00	37.0 ± 4.4	30.2 ± 4.1***	−1.57	35.5 ± 9.2	30.6 ± 7.6***	−0.50	*F* = 111.7; *p* < 0.001	*F* = 6.19; *p* = 0.008	*F* = 11.9; *p* < 0.001
MPF (N)	788.4 ± 119.8	710.1 ± 101.9^CS2***^	−0.69	781.5 ± 120.2	735.8 ± 108.0**	−0.39	783.9 ± 108.6	735.5 ± 98.9***	−0.46	*F* = 35.1; *p* = 0.001	*F* = 0.66; *p* = 0.005	*F* = 7.13; *p* = 0.005
MPV (m·s^−1^)	0.98 ± 0.08	0.83 ± 0.12^CS6***^	−1.44	0.98 ± 0.08	0.87 ± 0.10***	−1.19	0.98 ± 0.07	0.88 ± 0.10***	−1.06	*F* = 86.9; *p* < 0.001	*F* = 3.2; *p* = 0.06	*F* = 4.44; *p* = 0.27
MPP (w)	700.0 ± 118.1	550.6 ± 109.6^, CS6***^	−1.29	694.1 ± 118.9	594.6 ± 119.6***	−0.82	687.2 ± 104.8	592.7 ± 93.3***	−0.93	*F* = 61.2; *p* < 0.001	*F* = 1.4; *p* = 0.27	*F* = 7.1; *p* = 0.005
Neuromuscular response												
RMS (%)	123.8 ± 25.4	111.4 ± 30.1	−0.43	117.0 ± 33.4	94.9 ± 23.3**	−0.75	115.6 ± 29.8	107.3 ± 28.0	−0.28	*F* = 16.7; *p* = 0.001	*F* = 0.80; *p* = 0.47	*F* = 1.49; *p* = 0.26
MDF (%)	95.0 ± 9.2	92.6 ± 10.4	−0.24	97.6 ± 10.4	92.5 ± 13.9	−0.40	97.0 ± 7.7	91.6 ± 19.4	−0.35	*F* = 2.98; *p* = 0.11	*F* = 0.05; *p* = 0.95	*F* = 0.24; *p* = 0.79

Data are mean ± SD, *n* = 22. TS, traditional sets; CS6, cluster sets of 6 repetitions; CS2, cluster sets of 2 repetitions; CMJ, countermovement jump height; MPF, mean propulsive force; MPV, mean propulsive velocity; MPP, mean propulsive power; RMS, root mean square from electromyography (EMG) data; MDF, median frequency from EMG data. Both mechanical and neuromuscular data were obtained from the test against the load that elicited a ∼1 m·s^−1^ velocity at baseline measurements in full-squat. Pre, baseline measure; Post, after exercise; ES, effect size. Statistically significant differences with a CS2 protocol: CS2 (*p*<0.05). Statistically significant differences with a CS6 protocol: CS6 (*p*<0.05). Intragroup significant differences from Pre- to Post-training: ^*^p< 0.05 **p< 0.01, ***p< 0.001.

## 4 Discussion

The main findings of this study were: (1) CS demonstrated enhanced performance maintenance (i.e., MPF, MPV, and MPP) and exhibited reduced neuromuscular alterations (i.e., RMS and MDF) throughout repetitions compared to TS. (2) Post-exercise tests demonstrated that CS led to lesser impairments in mechanical performance (such as CMJ height, MPF, MPV, and MPP) and mitigated alterations in blood lactate and muscle contractile properties (Dm, Vd, and Tc) when contrasted with TS.

### 4.1 Changes in performance across repetitions and metabolic response

All protocols induced declines in performance parameters; however, adding more frequent rest periods between repetitions demonstrated superior performance maintenance while exhibiting less pronounced blood lactate responses. Consistent with our findings, previous research has noted enhanced performance maintenance by incorporating intra-set rests during squat (11, 38–40) and bench-press (41) exercises. The observed higher lactate concentrations with longer set configurations suggest an increased reliance on anaerobic glycolysis for energy production and an impaired replenishment of ATP and PCr stores (42, 43). It has been proposed that within-set rest intervals, characteristics of CS, facilitate the recovery of bioenergetic components like PCr and ATP (13). Consequently, longer set configurations may result in reduced PCr store maintenance, increased muscle metabolite levels, and partial ATP resynthesis (7, 42). These findings underscore the effectiveness of CS in mitigating fatigue development by minimizing metabolic by-product accumulation, thereby potentially enhancing the ability to sustain mechanical performance.

### 4.2 Changes in neuromuscular properties across repetitions

The preservation of mechanical performance may be linked to neuromuscular activity (44). Our findings reveal that more frequent rest periods between repetitions resulted in less neuromuscular fatigue during dynamic contractions, evidenced by lower RMS and higher MDF values, compared to TS. These findings align with Piqueras-Sanchiz et al. (21), noting lower MDF values in a 3 × 8 protocol compared to 6 × 4 at 75% 1RM in SQ. Similarly, Ortega-Becerra et al. (41) demonstrated that intra-rest periods of 30 s in CS protocols minimized neuromuscular fatigue and improved mechanical performance. Increased EMG amplitude (i.e., RMS) observed during longer set configurations may indicate greater muscle activation involving higher-threshold motor units, increased firing frequency, and changes in intrinsic muscle properties to compensate for force loss in the fatigued state (45, 46). Additionally, longer set configurations induced lower MDF values linked to decreased action potential conduction velocity, reduced firing rate of fatigued fast motor units (46), lowered intramuscular pH (47), and changes in action potential shape (48). Metabolic by-product accumulation could disrupt muscle function and impair neuromuscular signaling (45).

### 4.3 Changes in muscle contractile properties

Our study indicates that the CS methodology may mitigate the exercise-induced impairments in muscle twitch contractile responses, as evidenced by less pronounced alterations in TMG parameters for shorter set configurations. Regarding Vd, consistent with previous research (21), shorter set configurations were linked to lesser reductions in Vd. Reduced Vd may stem from decreased Dm and increases in Tc and Td (30, 31). Longer set configurations induced higher reductions in Dm and increases in Tc (Table 3). The decline in Dm observed following RT is associated with impaired muscle function (49), potentially influenced by exercise-induced muscle damage and muscle swelling (50). The longer post-exercise Tc observed for TS compared to CS2 reinforces the hypothesis of greater loss of muscle velocity of deformation following longer set configurations. However, unexpectedly, Td decreased for both TS and CS2, suggesting that the time to begin the contraction was shortened for both protocols. This has been attributed to improved neuromuscular coordination, increased muscle recruitment, and enhanced muscle fiber excitability (19). However, the causes behind why only the TS and CS2 protocols resulted in decreased Td are unknown. Further investigation is warranted to comprehensively explore this finding, and examine the underlying mechanisms and potential implications for training methodologies.

### 4.4 Changes in performance tests following the protocols

While past studies (10, 12) have primarily emphasized sustaining performance levels during RT sessions, limited knowledge exists about the residual fatigue resulting from different set configurations. Although no significant differences between protocols were observed for the isometric performance, our study revealed that more frequent rest periods between repetitions led to reduced residual fatigue in the dynamic tasks (i.e., lower loss of CMJ height, along with MPF, MPV, and MPP during the V1-load test). A recent review conducted by Jukic et al. (2) highlighted the potential of CS in mitigating residual fatigue induced by RT. However, it is noteworthy that while CS showed efficacy in reducing fatigue accumulated within RT sets, its impact on residual fatigue appeared comparatively lower (27). In this regard, a recent study (51) has shown that a CS protocol reduced fatigue within the set and resulted in quicker rates of recovery than the TS protocols. This approach holds practical relevance for strength and conditioning professionals, particularly in scenarios where competitions occur weekly or every 3–4 days. It enables athletes to recover faster and be better prepared for subsequent training sessions or competitions within a condensed timeframe.

In short, utilizing short and frequent intra-set rest intervals proved effective in sustaining mechanical performance, reducing metabolic stress, and mitigating alterations of neuromuscular markers of fatigue and muscle contractile properties. As such, CS emerges as an effective strategy to alleviate neuromuscular fatigue development and minimize the accumulation of metabolic by-products, ultimately enhancing the capacity to sustain mechanical performance.

Several limitations should be considered when interpreting our results. First, the TMG's maximum intensity may not evoke a maximal twitch for the participants. This may present problems for the fatigue-related deductions about the muscle. Second, EMG has been analyzed pooled in both muscles (i.e., VL and VM). Although this approach may represent the superficial vastii activity, the response may be slightly different if both muscles were analyzed separately. Future studies should examine the long-term effects of these protocols on mechanical, neuromuscular, and hypertrophic adaptations.

## 5 Practical applications

Strength and conditioning coaches should consider integrating short, frequent intra-set rest periods into the training sessions when fatigue development is undesired. This strategy maximizes force production, velocity, and power output while alleviating metabolic stress, neuromuscular fatigue, and adverse alterations in muscle contractile properties. This may be implemented during the in-season period when fatigue development is undesired. In time-constrained scenarios, splitting into two halves of the set, such as CS6, can effectively manage fatigue during and after RT without significantly extending the total training duration.

## 6 Conclusion

Utilizing short and frequent intra-set rest intervals proved effective in sustaining mechanical performance, reducing metabolic stress, and mitigating alterations of neuromuscular markers of fatigue and muscle contractile properties. As such, CS emerges as an effective strategy to alleviate neuromuscular fatigue development and minimize the accumulation of metabolic by-products, ultimately enhancing the capacity to sustain mechanical performance.

## Data Availability

The datasets presented in this study can be found in online repositories. The names of the repository/repositories and accession number(s) can be found below: https://data.mendeley.com/drafts/v6bgf8z547.
